# Interleukin-7 promotes porcine early embryogenesis in vitro and inner cell mass development through PI3K/AKT pathway after parthenogenetic activation

**DOI:** 10.1038/s41598-025-98574-z

**Published:** 2025-04-22

**Authors:** Dongjin Oh, Hyerin Choi, Mirae Kim, Ali Jawad, Joohyeong Lee, Byoung Chol Oh, Sang-Hwan Hyun

**Affiliations:** 1 Laboratory of Veterinary Embryology and Biotechnology (VETEMBIO), Veterinary Medical Center and College of Veterinary Medicine, Chungbuk National University, Cheongju, South Korea; 2 Institute of Stem Cell and Regenerative Medicine (ISCRM), Chungbuk National University, Cheongju, South Korea; 3 Department of Companion Animal Industry, Semyung University, Jecheon, 27136 Republic of Korea; 4 Department of Plastic and Reconstructive Surgery, Johns Hopkins University School of Medicine, Baltimore, MD USA; 5 Vet-ICT Convergence Education and Research Center (VICERC), Chungbuk National University, Cheongju, Republic of Korea; 6 Chungbuk National University Hospital, Cheongju, Republic of Korea

**Keywords:** Interleukin-7, Porcine early embryogenesis, PI3K/AKT signaling pathway, Inner cell mass, In vitro culture, Embryology, Cell lineage

## Abstract

Interleukin-7 (IL-7) plays a crucial role in cell survival and proliferation through the phosphatidylinositol-3-kinase (PI3K)/AKT signaling. While we previously demonstrated the beneficial role of IL-7 in early porcine embryonic development, the underlying molecular mechanisms remained unclear. We hypothesized that IL-7 would enhance early embryogenesis and promote inner cell mass (ICM) formation via PI3K/AKT pathway activation. To test this, embryos were cultured with wortmannin (Wort), a PI3K inhibitor, with or without IL-7 after parthenogenetic activation. IL-7 supplementation significantly increased cleavage and blastocyst formation rates compared to the control (*p* < 0.05), while mitigating Wort-induced developmental impairment. Moreover, IL-7 significantly reduced blastocyst apoptosis and increased total cell numbers compared to the control (*p* < 0.05), thereby counteracting pro-apoptotic effects of Wort. Furthermore, IL-7 treatment significantly promoted ICM formation through the PI3K/AKT pathway, as demonstrated by increased SOX2 + cell numbers and ICM-specific gene expression, with elevated phosphorylated AKT levels compared to the control (*p* < 0.05). Notably, IL-7 significantly improved mitochondrial function and biogenesis-related gene expression compared to the control (*p* < 0.05) through a PI3K/AKT-independent pathway. These findings suggest that IL-7-mediated PI3K/AKT signaling enhances porcine early embryonic development in vitro, providing insights into mechanisms that regulate early embryonic development in mammals.

**Supplementary Information:**

The online version contains supplementary material available at 10.1038/s41598-025-98574-z.

## Introduction

Assisted reproductive technologies, including gamete and embryo cryopreservation as well as in vitro fertilization (IVF), have been extensively employed in both livestock species and human reproductive medicine for several decades, effecting a revolution in breeding strategies and infertility treatment. In cattle, in vitro production (IVP) is well-established methodology, making a significant contribution to the optimization of industrial-scale animal production and genetic improvement strategies. Concurrently, it has facilitated notable advancements in both beef and dairy production^1,2^. Given the pivotal role of pigs in global meat consumption, constituting a substantial proportion of worldwide meat production^3^, it is imperative to enhance pig productivity through IVP techniques to ensure a consistent and sustainable pork supply for both developed and developing countries^4^.

Pigs display greater physiological and genetic similarities to humans than other livestock species, making them an invaluable research model in diverse scientific fields, including embryonic development, reproductive biotechnology, and the study of human diseases^5^. These properties have facilitated the integration of porcine embryo IVP with advanced gene editing techniques, enabling the development of various pig models for human-like diseases^6,7^, xenotransplantation^8,9^, and virus resistance to improve the swine industry^10,11^. Moreover, recent studies have reported the generation of interspecies chimeras containing human tissue through the complementation of porcine embryos with human pluripotent stem cells, as a solution for organ transplantation^12,13^. Despite these advancements, porcine IVP still exhibits a comparatively low success rate relative to other livestock species^14^. Furthermore, the quality of in vitro-produced embryos remains inferior to their in vivo-derived embryos^4^. It is therefore necessary to optimize in vitro culture (IVC) systems to enhance embryo quality and facilitate effective porcine embryonic development, thereby ensuring the successful generation of porcine models.

The inner cell mass (ICM) has the capacity to differentiate into all embryonic cell lineages, thus serving as a predictive biomarker of embryo quality through the assessment of the ICM ratio within the blastocyst^15^. Hence, a comprehensive understanding of the developmental dynamics and functional significance of the ICM is crucial for the optimization of porcine embryonic development in vitro. The application of RNA sequencing technologies has yielded substantial insights into the regulation of signaling pathways in the context of preimplantation embryonic development across a range of mammalian species^16–18^. Transcriptomic profiling has revealed that the predominant signaling pathways in the porcine ICM are the phosphatidylinositol-3-kinase (PI3K)/AKT and the Janus kinase–signal transducer and activator of transcription (JAK-STAT) pathways^17,19^. These pathways were found to be enriched in the ICM of porcine early blastocysts through Kyoto Encyclopedia of Genes and Genomes pathway analysis^17,19^, and treatment with specific pathway inhibitors during lineage segregation resulted in decreased porcine ICM cell numbers^17^. These findings indicate that these signaling pathways play a pivotal role in porcine ICM specification^19^. Although the importance of the PI3K/AKT signaling pathway in preimplantation embryonic development has been extensively investigated in mice^20–22^, its function in porcine early embryogenesis and lineage specification remains less well understood.

Interleukin-7 (IL-7), a key cytokine in lymphocyte development, regulates the survival and proliferation of these cells. Upon binding to its receptor, IL-7 activates JAK3 and JAK1^23^. Activated JAK1 recruits PI3K and STAT proteins. Subsequently, PI3K phosphorylates AKT, promoting anti-apoptotic pathways and cell survival^24^. IL-7 activates the PI3K/AKT signaling pathway, which is crucial for cell viability, proliferation, and metabolic activity in T cell acute lymphoblastic leukemia cells^25^. Our previous research demonstrated that IL-7 treatment enhances the expression of PI3K/AKT signaling-related genes in blastocysts and improves embryonic development and blastocyst quality in pigs^26^. However, the relationship between IL-7 and the activation of the PI3K/AKT pathway during early embryonic development in mammals remains unclear.

The objective of this study was to test the hypothesis that IL-7 would enhance early embryogenesis and promote ICM formation in porcine blastocysts through the activation of the PI3K/AKT pathway. Due to high polyspermy rates compared to other species, porcine IVF yields low blastocyst development rates^27^. Therefore, researchers frequently use parthenogenetic activation (PA) to investigate the mechanisms of porcine early embryonic development^17,28,29^. To test this hypothesis, we employed wortmannin (Wort), a PI3K inhibitor, to elucidate the relationship between IL-7 treatment and PI3K/AKT signaling during porcine IVC after PA. Specifically, we evaluated cleavage patterns, blastocyst formation rates, and blastocyst apoptosis, as well as mitochondrial content and membrane potential. Moreover, we validated ICM development by examining SOX2 + cells, a porcine ICM marker^30^, and the expression of PI3K/AKT signaling-related proteins through immunofluorescence, and analyzed lineage-specific gene expression in porcine blastocysts.

## Results

### IL-7 enhances porcine early embryonic development through the PI3K/AKT signaling pathway

**Fig. 1:**
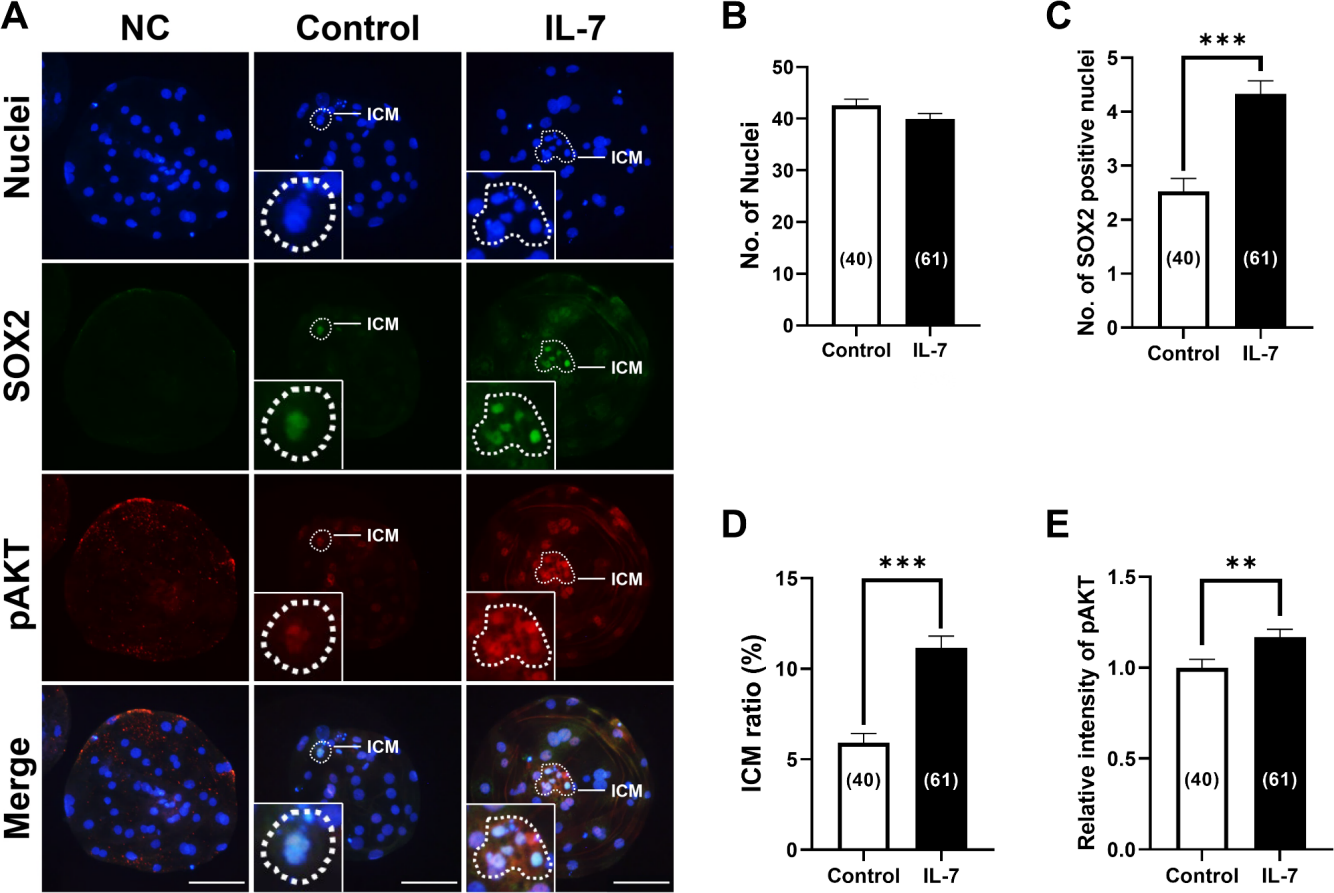
Effects of interleukin-7 (IL-7) treatment during in vitro culture (IVC) on inner cell mass (ICM) development and phosphorylated AKT (pAKT) expression of parthenote embryos. (**A**) Representative immunofluorescence images of porcine parthenote blastocysts labeled with Hoechst 33342 (Total nuclei, blue), SOX2 (ICM marker, green), and pAKT (red). Scale bar, 100 μm. Quantification of (**B**) the total cell number, (**C**) SOX2 positive cell number, (**D**) ICM ratio and (**E**) relative intensity of pAKT in the control and IL-7 treatment groups. Embryos were treated with IL-7 (10 ng/mL). The number of embryos examined in each experimental group is shown in parentheses. Asterisks indicate statistical significance (**p < 0.01 and ***p < 0.001). For all graphs, the values represent the mean ± SEM. The experiment was independently replicated four times. Statistical significance was determined using Student’s t-test.

We have previously demonstrated that IL-7 treatment during IVC increases the ICM ratio in porcine blastocysts and upregulates genes related to the PI3K/AKT pathway^26^. To investigate whether a correlation exists between the increased ICM in IL-7-treated blastocysts and the PI3K/AKT signaling, we examined the expression of SOX2 and phosphorylated AKT (pAKT) in blastocyst treated with IL-7 (Fig. 1A). Quantitative analysis revealed no significant difference in total cell numbers between IL-7-treated and control blastocysts (Fig. 1B). However, IL-7-treated blastocysts exhibited a significantly (*p* < 0.001) increased number of SOX2 + nuclei and overall ICM ratio compared to the control (Fig. 1C and D). Notably, pAKT displayed heterogeneous expression in both the nuclei and cytoplasm of ICM cells within porcine blastocysts. Moreover, pAKT expression was significantly (*p* < 0.01) elevated in IL-7-treated blastocysts compared to the control group (Fig. 1E).

**Fig. 2:**
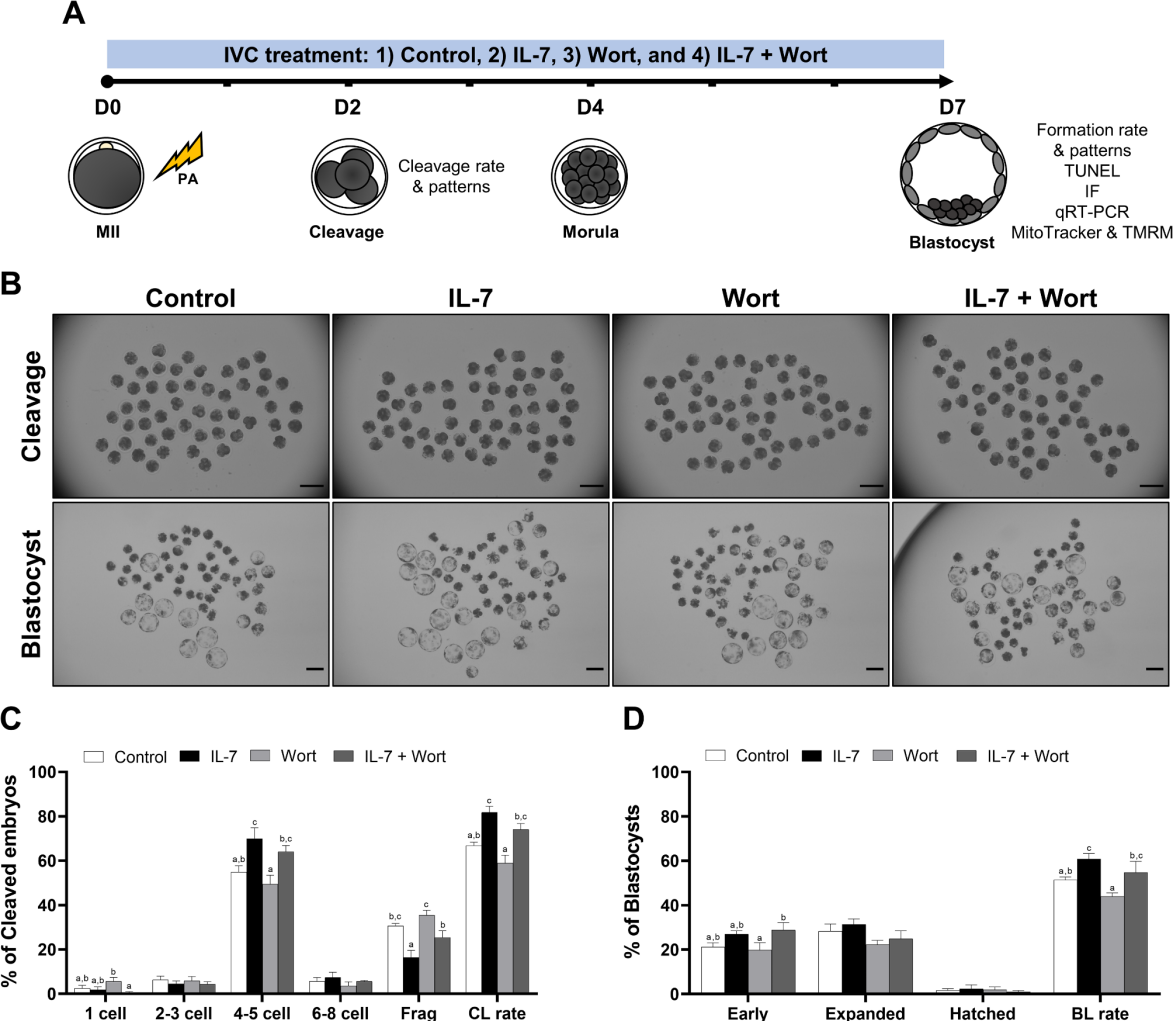
Effect of IL-7 and wortmannin (Wort) treatment during IVC for 7 days on early porcine embryonic development after parthenogenetic activation (PA). (**A**) Experimental design to examine the effects of IL-7 and the phosphatidylinositol 3-kinase (PI3K)/AKT signaling pathway on porcine early embryogenesis and ICM development after PA. (**B**) Representative morphology images of cleaved embryos after Day 2 of PA and blastocysts from each group after Day 7 of PA. Scale bar, 200 μm. (**C**) The cleavage pattern and (**D**) blastocyst formation rates of the PA embryos. Embryos were treated with IL-7 (10 ng/mL) and/or Wort (1 µM). The number of cultured embryos in each group was Control (*n* = 197), IL-7 (*n* = 200), Wort (*n* = 195), and IL-7 + Wort (*n* = 199). Within each end point, bars with different letters (a-c) are significantly different (*p* < 0.05) among groups. For all graphs, the values represent the mean ± SEM. The experiment was independently replicated five times. Frag, Fragmentation; CL, Cleavage; BL, Blastocyst.

To elucidate the interaction between IL-7 and the PI3K/AKT pathway during porcine early embryonic development, we utilized Wort, a PI3K inhibitor, during IVC following PA (Fig. 2A). Wort treatment induced a time-dependent decrease in pAKT levels in porcine embryos cultured in vitro (Supplementary Fig. 1), consistent with previous reports in various cell types^31,32^. In the absence of Wort, IL-7 supplementation significantly (*p* < 0.05) enhanced both cleavage and blastocyst formation rates compared to the control group (Fig. 2B). Conversely, Wort treatment significantly (*p* < 0.05) reduced cleavage and blastocyst formation rates, and significantly (*p* < 0.05) increased embryo fragmentation compared to the IL-7 group (Fig. 2C and D). Notably, IL-7 supplementation mitigated the detrimental effects of Wort on porcine embryonic development (Fig. 2C and D).

### IL-7 regulates apoptosis in porcine embryos through the PI3K/AKT signaling pathway

**Fig. 3:**
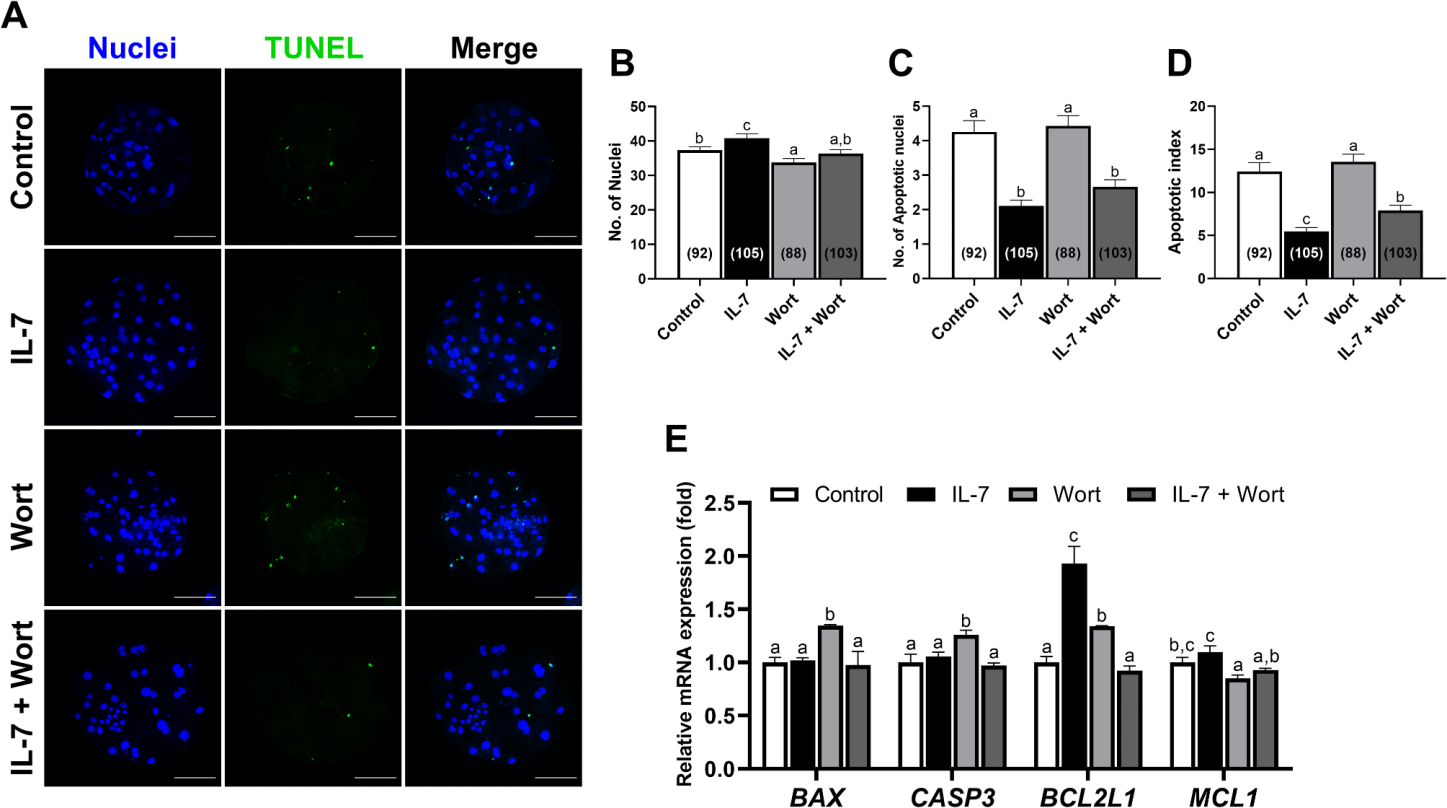
Numbers of total cells and apoptotic nuclei in PA-derived blastocysts exposed to IL-7 and Wort treatment during IVC for 7 days. (**A**) Representative laser scanning confocal microscopy images of porcine blastocysts labeled with Hoechst 33342 (Total nuclei, blue) and TUNEL (Apoptotic nuclei, green). Scale bar, 100 μm. Quantification of (**B**) the total cell number, (**C**) apoptotic cell number, and (**D**) apoptotic index in the indicated groups. The number of embryos examined in each experimental group is shown in parentheses. The TUNEL assay was independently replicated four times. (**E**) Quantification of mRNA expression of apoptosis‑related genes in blastocysts from each group by qRT-PCR. Data were normalized to the *RN18S* gene. Embryos were treated with IL-7 (10 ng/mL) and/or Wort (1 µM). Within each endpoint, bars with different letters (a-c) are significantly different (*p* < 0.05) among groups. For all graphs, the values represent the mean ± SEM. The qRT-PCR was independently replicated three times.

Our previous research demonstrated that IL-7 supplementation exerts an anti-apoptotic effect on porcine blastocysts^26^. To determine whether these anti-apoptotic effects are mediated via the PI3K/AKT pathway in porcine blastocysts, we assessed blastocyst apoptosis using terminal deoxynucleotidyl transferase-mediated dUTP nick end labeling (TUNEL) assay (Fig. 3A). IL-7-treated blastocysts exhibited a significant (*p* < 0.05) increase in total cell number compared to other groups (Fig. 3B). Notably, Wort treatment led to a significant (*p* < 0.05) reduction in total cell number compared to the control, while the IL-7 + Wort group restored cell numbers to levels comparable with control blastocysts (Fig. 3B). The number of apoptotic nuclei in blastocysts was significantly (*p* < 0.05) reduced in the IL-7-treated groups compared to the other groups (Fig. 3C). Similarly, the apoptotic index was most significantly (*p* < 0.05) reduced in IL-7-treated blastocysts compared to control, followed by the IL-7 + Wort group (Fig. 3D). Consistent with these results, mRNA expression analysis revealed that the apoptosis-related genes *BAX* and *CASP3* were significantly (*p* < 0.05) upregulated in the Wort group compared to the control, whereas the IL-7 + Wort group showed no significant (*p* > 0.05) different from the control (Fig. 3E). Expression of anti-apoptotic genes *BCL2L1* and *MCL1* was significantly (*p* < 0.05) higher in the IL-7-treated group compared to the Wort group (Fig. 3E).

### IL-7 enhances ICM development in porcine blastocysts through the PI3K/AKT signaling pathway

**Fig. 4:**
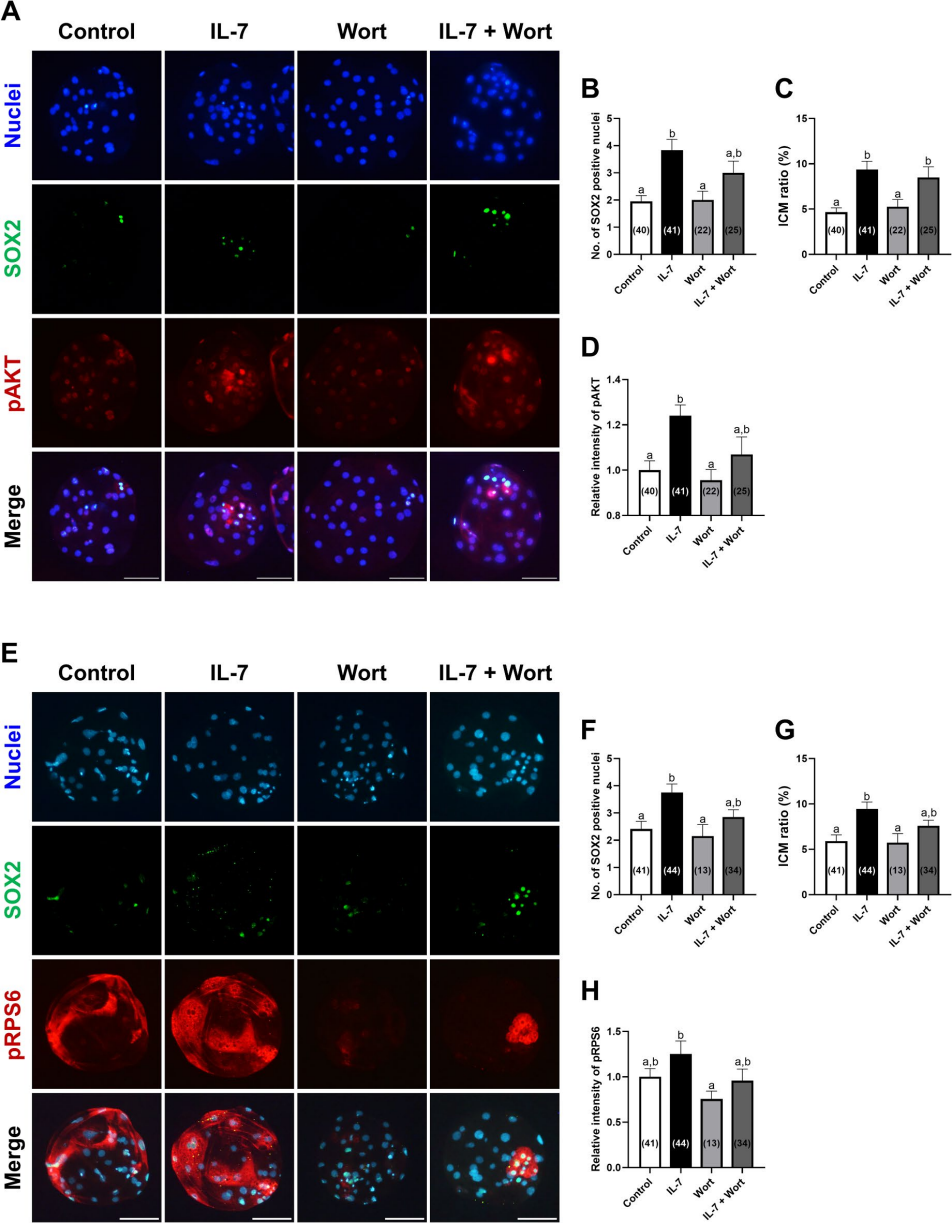
Effects of IL-7 and Wort treatment during IVC for 7 days on ICM development, pAKT, and phosphorylated ribosomal protein S6 (pRPS6) expression of parthenote embryos. (**A**) Representative immunofluorescence images of porcine parthenote blastocysts labeled with Hoechst 33342 (Total nuclei, blue), SOX2 (ICM marker, green), and pAKT (red). Scale bar, 100 μm. Quantification of the (**B**) SOX2 positive cell numbers, (**C**) ICM ratio and (**D**) relative intensity of pAKT in the indicated groups. (**E**) Representative immunofluorescence images of porcine parthenote blastocysts labeled with Hoechst 33342 (Total nuclei, blue), SOX2 (green), and pRPS6 (red). Scale bar, 100 μm. Quantification of the (**F**) SOX2 positive cell numbers, (**G**) ICM ratio and (**H**) relative intensity of pRPS6 in the indicated groups. Embryos were treated with IL-7 (10 ng/mL) and/or Wort (1 µM). The number of embryos examined in each experimental group is shown in parentheses. Within each endpoint, bars with different letters (a, b) are significantly different (*p* < 0.05) among groups. For all graphs, the values represent the mean ± SEM. The experiment was independently replicated four times.

Next, we examined the expression of porcine ICM markers SOX2 and pAKT in each group to determine whether IL-7 treatment modulates ICM formation via the PI3K/AKT pathway in porcine blastocysts (Fig. 4A). Notably, pAKT exhibited enhanced cytoplasmic expression within the ICM of blastocyst treated with IL-7 (Fig. 4A). IL-7-treated blastocysts showed a significant (*p* < 0.05) increase in SOX2 + cell numbers compared to both control and Wort-treated groups, while the combined treatment of IL-7 and Wort showed no significant (*p* > 0.05) difference in SOX2 + cells compared to the IL-7 group (Fig. 4B). The ICM ratio was significantly (*p* < 0.05) increased in IL-7 treated groups compared to other groups (Fig. 4C). Quantification of pAKT levels in blastocyst revealed that IL-7 supplementation significantly (*p* < 0.05) increased pAKT levels compared to the control, an effect that was reduced by Wort co-treatment (Fig. 4D).

To further validate PI3K/AKT activation, we assessed the expression levels of phosphorylated ribosomal protein S6 (pRPS6), a well-established downstream target and widely utilized readout of PI3K/AKT pathway activity^33^. Consistent with the pAKT expression patterns, pRPS6 displayed heterogeneous cytoplasmic expression in porcine blastocysts (Fig. 4E). SOX2 levels and ICM ratio in blastocysts were significantly (*p* < 0.05) higher in the IL-7 group, with these enhancements attenuated by Wort co-treatment (Fig. 4F and G). In parallel, IL-7-treated blastocysts showed significantly (*p* < 0.05) higher pRPS6 levels compared to the Wort group, while no significant (*p* > 0.05) difference was observed in the IL-7 + Wort group (Fig. 4H).

**Fig. 5:**
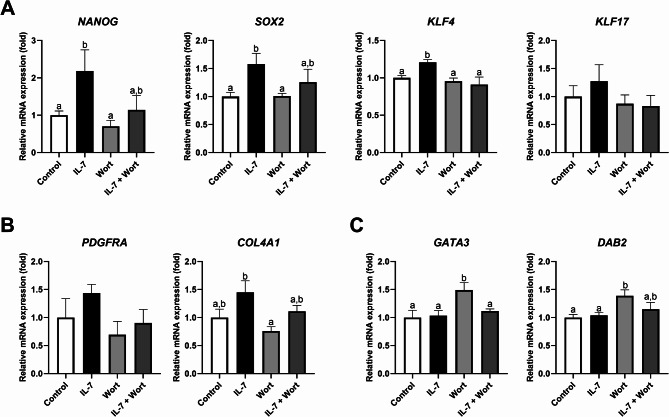
Effects of IL-7 and Wort treatment during IVC for 7 days on mRNA expression levels of lineage-specific genes. The mRNA expression levels of (**A**) epiblast markers (*NANOG*, *SOX2*, *KLF4*, and *KLF17*), (**B**) hypoblast markers (*PDGFRA* and *COL4A1*), and (**C**) trophectoderm markers (*GATA3* and *DAB2*) at blastocysts from each group. Embryos were treated with IL-7 (10 ng/mL) and/or Wort (1 µM). Within each endpoint, bars with different letters (a, b) are significantly different (*p* < 0.05) among groups. Data were normalized to the *RN18S* gene. For all graphs, the values represent mean ± SEM. The experiment was independently replicated four times.

Furthermore, we confirmed the expression of previously reported genes related to lineage specification in porcine blastocysts^17^. Consistent with the SOX2 protein expression results, the mRNA expression levels of the ICM markers *NANOG*, *SOX2*, and *KLF4* were significantly (*p* < 0.05) higher in blastocysts treated with IL-7 compared to the control and Wort groups, while the IL-7 + Wort treatment group showed no significant (*p* > 0.05) difference (Fig. 5A). In addition, the hypoblast-related gene *COL4A1* were significantly (*p* < 0.05) increased in the IL-7-treated group compared to the Wort group (Fig. 5B). Conversely, trophectoderm (TE) markers *GATA3* and *DAB2* were significantly (*p* < 0.05) upregulated in the Wort group compared to the control (Fig. 5C).

### IL-7 enhances mitochondrial function in Porcine blastocysts through the PI3K/AKT- independent signaling pathway

**Fig. 6:**
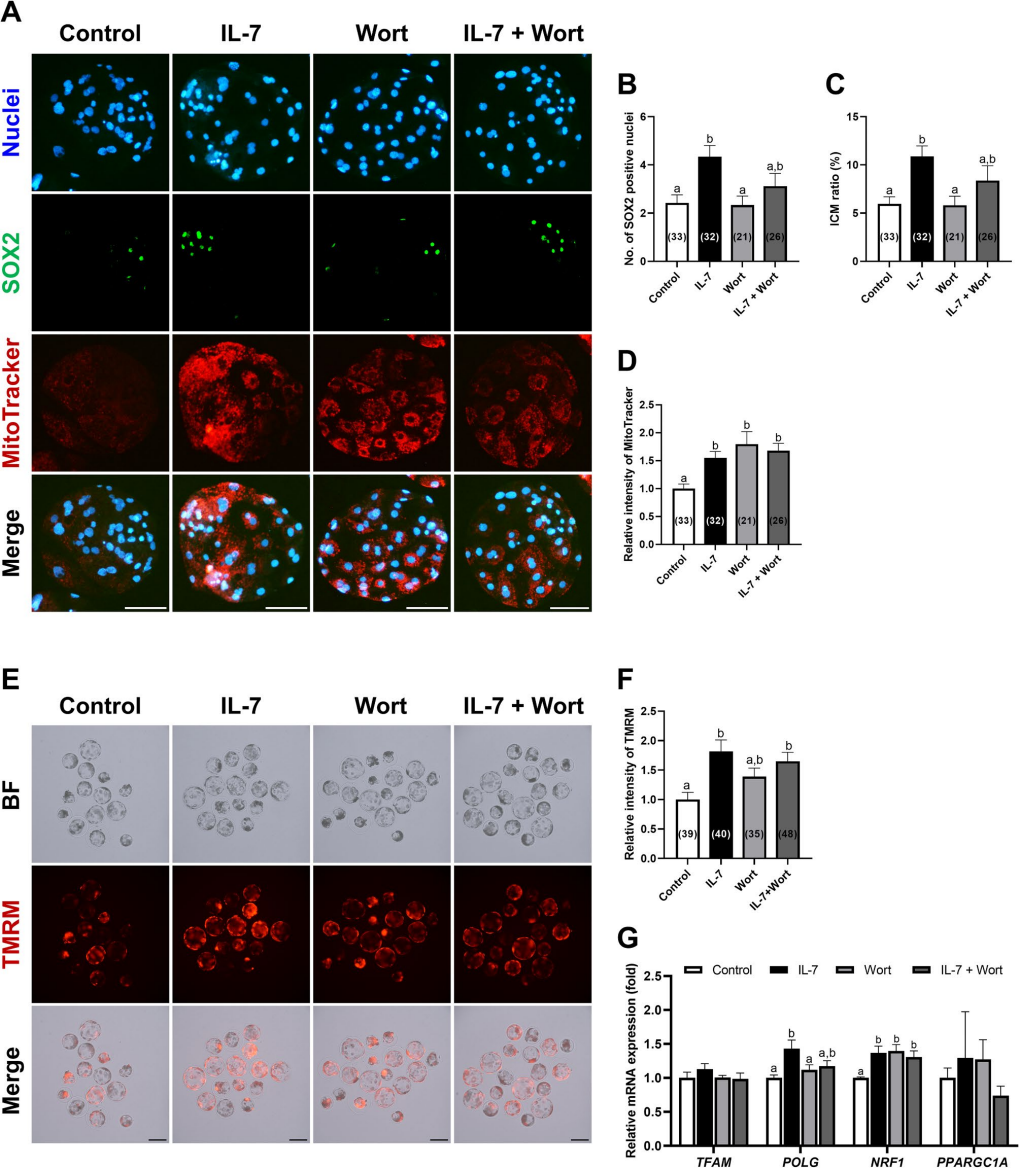
Effects of IL-7 and Wort treatment during IVC for 7 days on mitochondrial function. (**A**) Representative fluorescence images of porcine parthenote blastocysts labeled with Hoechst 33342 (Total nuclei, blue), SOX2 (ICM marker, green), and MitoTracker Red CMXRos staining (red). Scale bar, 100 μm. Quantification of the (**B**) SOX2 positive cell numbers, (**C**) ICM ratio and (**D**) relative intensity of MitoTracker in the indicated groups. (**E**) Representative TMRM fluorescence images of porcine parthenote blastocysts from each group. Scale bar, 200 μm. (**F**) Quantification of the relative intensity of TMRM in the indicated groups. The MitoTracker and TMRM assays were independently replicated three times. (**G**) Quantification of mRNA expression of mitochondrial biogenesis-related genes in blastocysts from each group by qRT-PCR. Data were normalized to the *RN18S* gene. Embryos were treated with IL-7 (10 ng/mL) and/or Wort (1 µM). Within each endpoint, bars with different letters (a, b) are significantly different (*p* < 0.05) among groups. For all graphs, the values represent the mean ± SEM. The qRT-PCR was independently replicated four times.

IL-7 regulates mitochondrial integrity, homeostasis, and respiratory chain via the PI3K/AKT pathway^25,34^. We thus investigated whether IL-7 treatment during IVC affects mitochondrial function through this pathway. To determine mitochondrial content in blastocysts, we utilized MitoTracker staining while simultaneously assessing SOX2 expression (Fig. 6A). Consistent with previous results, blastocysts treated with IL-7 exhibited significantly (*p* < 0.05) higher SOX2 levels and ICM ratios compared to the control, and these effects were attenuated by Wort treatment (Fig. 6B and C). Interestingly, MitoTracker intensity was significantly (*p* < 0.05) increased in all IL-7-treated groups, as well as in Wort-treated blastocysts compared to the control (Fig. 6D).

Next, we used TMRM, a cell-permeant label that accumulates in active mitochondria with intact membrane potential, to assess mitochondrial membrane potential in blastocysts (Fig. 6E). IL-7-treated blastocysts showed significantly (*p* < 0.05) higher TMRM intensity compared to the control group, consistent with MitoTracker staining results, and this enhancement persisted despite Wort co-treatment (Fig. 6F). In contrast, the Wort-treated group exhibited no significant (*p* > 0.05) difference in mitochondrial membrane potential from the control group (Fig. 6F). We also analyzed the expression levels of key mitochondrial biogenesis-related genes (*TFAM*, *POLG*, *NRF1*, and *PPARGC1A*) in each blastocyst. The expression levels of *TFAM* and *PPARGC1A* did not differ significantly (*p* > 0.05) among groups, whereas *POLG* expression was significantly (*p* < 0.05) elevated in the IL-7-treated group compared to the control (Fig. 6G). Notably, *NRF1* expression was significantly (*p* < 0.05) upregulated in all treatment groups compared to the control, aligning with our MitoTracker analysis results (Fig. 6G).

## Discussion

**Fig. 7:**
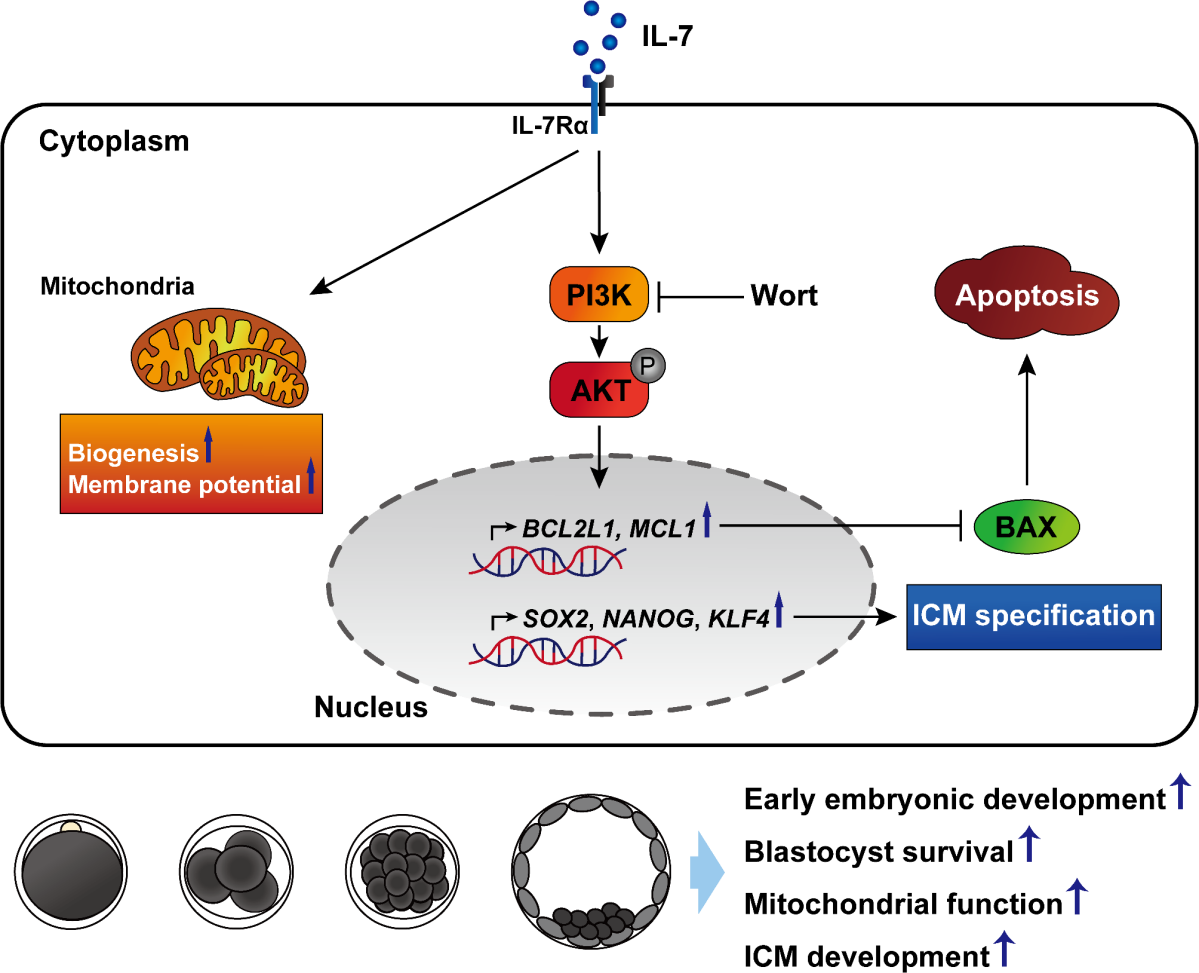
Schematic diagram illustrating the beneficial effects of IL-7 during porcine early embryonic development. IL-7 supplementation during IVC increases embryonic development and blastocyst survival through the PI3K/AKT pathway in porcine parthenote embryos. Moreover, IL-7 enhances mitochondrial function via a PI3K/AKT-independent pathway. Notably, IL-7-mediated activation of the PI3K/AKT signaling pathway enhances ICM development in porcine blastocysts by regulating ICM-specific gene expression.

The objective of this study was to determine whether IL-7 supplementation during IVC affects embryonic and ICM development via the PI3K/AKT pathway in porcine embryos. Our findings reveal that IL-7 plays a crucial role in porcine preimplantation embryos by regulating embryonic development and mitigating blastocyst apoptosis through the PI3K/AKT signaling pathway. Furthermore, IL-7 was observed to enhance mitochondrial function in porcine blastocysts. Additionally, it was demonstrated that IL-7 treatment activates proteins in the PI3K/AKT pathway, while concurrently upregulating the expression of the ICM marker SOX2 and its associated genes in blastocysts (Fig. 7).

The PI3K/AKT pathway plays a critical role in the proliferation and growth of a variety of cell types and is activated at all stages of the preimplantation embryos^21,35^. In a previous report, it was shown that pSer473-Akt not only localizes to the nucleus during major zygotic genome activation (ZGA), but also induces developmental arrest at the two-cell stage upon specific Akt inhibition in murine embryos^36^. We have previously demonstrated that IL-7 facilitates the development of blastocyst stage in porcine embryos by increasing the expression of genes related to ZGA and PI3K/AKT pathway^26^. Furthermore, the inhibition of PI3K/AKT pathway during the early stages of embryonic development has been shown to impair blastocyst development in mice, cattle, and pigs^20,37–39^. Consistent with previous studies, our results revealed that Wort-treated embryos exhibited a tendency to reduce cleavage rates and impede blastocyst formation. Notably, these deleterious effects were effectively mitigated through the IL-7 treatment. In addition, IL-7 supplementation was observed to enhance AKT phosphorylation and reverse the diminished phosphorylation levels induced by PI3K inhibition. Collectively, these findings suggest a potential function of IL-7 through PI3K/AKT signaling pathway in porcine embryonic development.

The PI3K/AKT pathway, a critical mediator of cellular survival mechanisms, exerts direct inhibitory effects on pro-apoptotic proteins such as BAX and caspase family members^40^. Inhibition of this pathway has been demonstrated to induce apoptosis in various cell types, including embryonic stem cells (ESCs), trophoblast stem cells (TSCs), and blastocysts^37,41,42^. Consistently, our results showed that Wort treatment induced the upregulation of pro-apoptotic genes, *BAX* and *CASP3*, resulting in increased fragmentation during embryonic development, a significant reduction in total cell number, and elevated apoptotic incidence in blastocysts. Conversely, IL-7 has been studied for its anti-apoptotic properties, which are mediated through modulation of BCL-2 family proteins in both precursor B cells^43^ and mature T lymphocytes^44^. Our previous investigation elucidated that IL-7 supplementation attenuates apoptosis in porcine blastocysts via regulation of apoptosis-associated genes^26^. This anti-apoptotic effect of IL-7 has been observed in various cellular contexts. For example, it has been demonstrated to counteract dexamethasone-induced cell death in mature T cells through PI3K-dependent signaling^45^ and to enhance murine thymocyte viability by inactivating the apoptotic factor Bad, a process partially mediated by PI3K activation^46^. Additionally, IL-7 has been shown to inhibit apoptosis in mouse granulosa cells through activation of the PI3K/AKT pathway^47^. Based on these findings, the current study demonstrated that IL-7 counteracted PI3K inhibition-induced apoptosis in porcine blastocysts and modulated apoptosis-related gene expression levels, suggesting that IL-7 enhances in vitro development by inhibiting apoptosis in porcine embryos, potentially through the PI3K/AKT pathway.

Interestingly, IL-7 treatment affected the increase of SOX2 + cells within porcine blastocysts. SOX2 has been established as a crucial transcription factor in diverse mammalian embryos, along with OCT4 and NANOG^16,17^. Furthermore, SOX2 is a reliable marker for the ICM in porcine preimplantation embryos and may be a key gene during first lineage specification^19,30^. Importantly, the functional interplay between SOX2 and the PI3K/AKT signaling pathway has been extensively documented across various malignant and stem cell populations^48,49^. Specifically, AKT activity primarily enhances ESC self-renewal by regulating Sox2 protein stability^50,51^. Inhibition of the PI3K/AKT pathway results in a reduction of SOX2 protein levels in breast carcinoma^52^ and esophageal squamous cells^53^, consistent with a previous finding in porcine embryos^17^. Additionally, a recent study demonstrated that PI3K signaling inhibition impairs ICM formation in mouse embryos, validating its role as a crucial regulator of mouse ICM fate acquisition^22^. In this study, IL-7 treatment increased PI3K/AKT-related protein levels and the ICM ratio in porcine blastocysts, and these effects were reduced by PI3K inhibition. Concordantly, studies have demonstrated that treatment with insulin^54^ or insulin growth factor 1^55^ enhances ICM proliferation in embryos through activation of the PI3K/AKT pathway. Collectively, these findings suggest that IL-7 enhances ICM development in porcine blastocysts via the PI3K/AKT/SOX2 signaling cascade.

Increased expression of pluripotency-associated genes, including *SOX2*, *NANOG*, and *KLF4*, is observed in the porcine ICM relative to the TE^17,19^. AKT has been reported to directly phosphorylate these transcription factors, thereby regulating their stability and activity^50,56^. Our results showed that IL-7 treatment upregulated the mRNA expression levels of *SOX2*, *NANOG*, and *KLF4* in blastocysts, whereas PI3K inhibition attenuated this IL-7-induced upregulation. Conversely, the TE markers *GATA3* and *DAB2* exhibited upregulation in the Wort-treated group, aligning with a previous report demonstrating that PI3K inhibition enhances the expression of key marker genes and promotes the formation of homogeneous colonies in TSCs^57^. Collectively, our findings indicate that IL-7 enhances the development of porcine ICM and upregulates the expression of associated transcription factors through activation of the PI3K/AKT signaling pathway.

Mitochondria play a pivotal role in energy production and maintenance of metabolic homeostasis during embryonic development^58,59^. The entire mitochondrial mass is crucial for generating the energy required by the embryo, with mitochondrial membrane potential serving as a key indicator of mitochondrial function^59^. Notably, mitochondria exhibiting high membrane potential correlate strongly with enhanced embryonic developmental potential^60^. Intriguingly, IL-7 has been reported to exert a significant influence on augmenting mitochondrial content and biogenesis in memory T cells^61^. Our results revealed that IL-7 supplementation markedly enhanced both mitochondrial mass and membrane potential in porcine blastocysts. Furthermore, we observed significantly elevated expression of mitochondrial biogenesis-associated genes, *POLG* and *NRF1*, in IL-7-treated blastocysts, consistent with previous observations in porcine and ovine oocyte studies^62,63^. Interestingly, PI3K inhibition neither adversely affected mitochondrial function in blastocysts during porcine embryonic development nor impacted blastocysts co-treated with IL-7. This observation suggests that IL-7 treatment enhances mitochondrial function in porcine embryos through a mechanism independent of the PI3K/AKT pathway. Collectively, these results indicate that IL-7 promotes pig embryonic development by enhancing mitochondrial function. However, elucidation of the precise underlying mechanisms necessitates further investigation.

Our results demonstrated that the beneficial effects of IL-7 supplementation during IVC are mediated by the PI3K/AKT signaling pathway during early porcine embryonic development. Specifically, IL-7 enhanced embryonic development, mitigated blastocyst apoptosis, and promoted ICM formation in porcine blastocysts via the PI3K/AKT pathway. Furthermore, IL-7 treatment enhanced mitochondrial function in porcine embryos, a process that was found to be independent of PI3K/AKT signaling. While these findings demonstrate several positive effects of IL-7, several points should be noted when interpreting these results. First, we used a single blocker (Wort) to investigate whether the positive effects of IL-7 on porcine early embryonic development are related to the PI3K/AKT pathway. Previous studies have observed that Wort can affect not only the PI3K/AKT pathway but also other pathways such as DNA-PK, ATM, ATR, and mTOR^64^. Therefore, additional validation using other PI3K/AKT-specific inhibitors or siRNA may be necessary. Since the effects of different inhibitors and their optimal concentrations on porcine early embryogenesis are unknown except for Wort^37^, further research is needed. Nevertheless, our results show that Wort could reduce IL-7-induced PI3K/AKT pathway activation in porcine blastocysts, providing important preliminary evidence for PI3K/AKT pathway involvement in IL-7-enhanced porcine early embryonic development and ICM formation. Second, we examined the effects of IL-7 during porcine early embryogenesis using only PA-derived embryos. Since parthenogenesis does not occur naturally in mammals, additional investigation of the effects of IL-7 during development of porcine IVF or somatic cell nuclear transfer-derived embryos is necessary. Third, regarding our experimental methodology, this study primarily utilized immunofluorescence analysis. For limited samples such as embryos, immunofluorescence provides several advantages including visualization of specific protein localization, cost-effectiveness, and the capability for multiple protein labeling. However, this approach has inherent limitations, such as quantification constraints and the possibility of non-specific binding, warranting validation using diverse methodological approaches. Finally, we examined only the PI3K/AKT pathway among various IL-7-mediated signaling pathways. Wort treatment alone could not completely inhibit the effects of IL-7, suggesting the involvement of other IL-7-mediated signaling pathways. Previously, IL-7 has been reported to activate other pathways that could affect embryo development, such as JAK/STAT and MAPK signaling pathways in lymphocytes and T-cell acute lymphoblastic leukemia cells^24,65^. Therefore, further investigation is needed to determine whether IL-7 influences porcine embryonic development through pathways beyond PI3K/AKT signaling. Despite these limitations, these findings provide new insights into the mechanisms by which IL-7 modulates porcine preimplantation embryo development and may contribute to advancements in porcine IVP systems and associated biotechnologies.

## Methods

### Chemicals

Unless otherwise indicated, all the chemicals and reagents used in this study were purchased from Sigma-Aldrich (Burlington, MA, USA).

### Oocyte collection and in vitro maturation (IVM)

Cumulus oocyte complexes (COCs) were retrieved from 3 to 7 mm follicles of ovaries from a local abattoir. COCs were washed twice with HEPES-buffered Tyrode’s medium containing 0.05% (w/v) polyvinyl alcohol (TLH-PVA). Then, COCs with intact cumulus cell layers and evenly granulated cytoplasm were selected for maturation and cultured in a four-well dish containing 500 µL IVM medium^66^ at 39 °C in 5% CO_2_ atmosphere. The IVM process consisted of the first 22 h of incubation in IVM medium containing 10 IU/mL equine chorionic gonadotropin and 10 IU/mL human chorionic gonadotropin, followed by 20 h of culture in hormone-free IVM medium. After IVM, the cumulus cells that surround the oocyte were removed by mechanical pipetting in the presence of 0.1% hyaluronidase for 1 min. Matured oocytes were determined by the presence of a polar body and used for further experiments.

### PA and IVC

Matured oocytes were washed thrice in calcium-free TLH-PVA, and then washed twice with 280 mM mannitol solution containing 0.01 mM CaCl_2_ and 0.05 mM MgCl_2_ for activation. The matured oocytes were placed in activation medium (260 mM mannitol solution containing 0.001 mM CaCl_2_ and 0.05 mM MgCl_2_), and subsequently activated using an Electro Cell Fusion Generator (LF101; Nepa Gene, Chiba, Japan) with two direct electrical pulses of 120 V/mm for 60 µs. Electro-activated oocytes were cultured in the IVC medium (porcine zygote medium (PZM)-3) containing 5 µg/mL of cytochalasin B for 4 h. Thereafter, PA embryos were washed thrice in fresh IVC medium and incubated in droplets of IVC medium covered with mineral oil at 39 °C in a humidified atmosphere of 5% CO_2_, 5% O_2_, and 95% N_2_. The embryos were transferred to fresh medium droplets containing fresh treatments (IL-7 and/or Wort) at 48 h (day 2) and 96 h (day 4) after PA. Cleavage and blastocyst formation were evaluated on day 2 and 7, respectively. During the entire IVC period, the IVC medium was treated with 0.01% (v/v) DMSO as a control group or 10 ng/mL IL-7 (200-07; Peprotech, Cranbury, NJ, USA), 1 µM Wort (W3144), and IL-7 + Wort as treated groups. The concentration and duration of IL-7 treatment were determined based on our previous study^26^, which showed optimal effects on embryonic development. The concentration of Wort was selected according to a previous study^37^, as this concentration inhibited PI3K/AKT signaling without affecting total cell numbers in blastocysts.

### Immunofluorescence

The blastocysts on day 7 were fixed with 4% paraformaldehyde (PFA) in PBS for 30 min at 25 °C (Room temperature; RT), washed twice in 0.1% PBS-PVA (PVS), and permeabilized in 0.5% Triton X-100 for 30 min at RT. After washing twice with 0.1% PVS, the blastocysts were blocked in blocking solution (0.1% PVS with 3% BSA) for 1 h at RT. Then, Blastocysts were incubated overnight at 4 °C with primary antibodies diluted in antibody dilution buffer (PBS with 1% BSA and 0.1% Tween 20). After washing thrice in 0.1% Tween 20 in 0.1% PVS (PVST) at RT, blastocysts were incubated for 1 h at RT with the appropriate secondary antibodies in antibody dilution buffer, followed by three washing for 10 min in PVST. Finally, the blastocysts were counterstained with Hoechst-33342 for 10 min and mounted on clean glass slides in an anti-fade mounting medium (S36937; Invitrogen, Carlsbad, CA, USA). The fluorescence intensities of stained proteins were detected using an epifluorescence microscope (TE300; Nikon). Quantitative image analysis was performed using the ImageJ software (http://imagej.nih.gov). SOX2-positive cells were specifically assessed in blastocysts with more than 30 cells and were counted only when co-localized with Hoechst staining. For cytoplasmic pAKT or pRPS6 in blastocysts, individual blastocysts were defined as regions of interest (ROI), and quantification was performed using the following formula: ROI integrated density-(ROI area * background mean), followed by normalization to control embryos. The antibodies used in this study are detailed in Supplementary Table 1.

### TUNEL assay

To determine number of apoptotic cells, blastocysts were stained with TUNEL using an in situ cell death detection kit (Roche, Basel, Switzerland). Blastocysts on day 7 washed twice in 0.1% PVS, fixed in 4% PFA in PBS for 1 h at RT, and washed twice in 0.1% PVS with 0.1% Tween 20 and 0.01% Triton X-100. The blastocysts were permeabilized by incubation with 0.3% TritonX-100 in PBS for 1 h at 37 °C, and then incubated with fluorescein-conjugated dUTP and terminal deoxynucleotidyl transferase for 1 h 30 min at 37 °C. Next, the blastocysts were washed twice with 0.1% PVS, counterstained with 5 µg/mL Hoechst-33342 for 10 min at RT, and mounted on clean glass slides in an anti-fade mounting medium. The total number of nuclei and apoptotic nuclei were detected under a confocal laser-scanning microscope (Zeiss LSM-880 with Airyscan). The number of apoptotic nuclei was counted based on the co-localization of TUNEL and Hoechst-33342 staining. The apoptotic index was calculated as (number of TUNEL-positive cells / total number of cells per blastocyst) × 100.

### Quantitative reverse transcription-polymerase chain reaction (qRT-PCR)

Blastocysts were washed three times with 0.1% PVS and RNA was extracted using TRIzol reagent (TaKaRa Bio, Inc., Otsu, Shiga, Japan) according to the manufacturer’s protocol. Subsequently, the extracted RNA (1 µg of total RNA) was converted to complementary DNA (cDNA) using SuperScript IV VILO Master Mix (Thermo Fisher Scientific, Waltham, MA, USA). The synthesized cDNA, which was used as the template, 2× SYBR Premix Ex Taq (TaKaRa Bio, Inc.) and 5 pmol of specific primers (Macrogen, Inc., Seoul, Republic of Korea) were used for qRT-PCR. The mRNA expression was carried out at 95 °C for 5 min, followed by 40 cycles of 95 °C for 15 s, 56 °C for 15 s, and 72 °C for 30 s using the CFX96 Touch Real-Time PCR Detection System (Bio-Rad, Hercules, CA, USA). Relative quantification was normalized to *RN18S*^67^ and calculated by comparing the threshold cycle (Ct) at constant fluorescence intensity. Relative mRNA expression (R) was performed using the equation *R* = 2^−[ΔCtsample−ΔCtcontrol]^^68^. All primer sequences are listed in Supplementary Table 2.

### Measurement of mitochondrial distribution and membrane potential

To investigate the distribution of active mitochondria, blastocysts were incubated with 500 nM MitoTracker Red CMXRos (M7512; Invitrogen) at 39 °C for 30 min. After washing thrice with IVC medium, the blastocysts were stained as described in “Immunofluorescence”. To examine the mitochondrial membrane potential, blastocysts incubated with 200 nM tetramethylrhodamine, methyl ester (TMRM; T668; Invitrogen) for 30 min. After washing twice with 0.1% PVS, the fluorescence intensities of TMRM were detected using an epifluorescence microscope (TE300; Nikon). Quantitative image analysis was performed using the ImageJ software.

### Western blot analysis

Forty embryos were washed twice in 0.1% PVS and subsequently lysed in RIPA buffer (89900; Thermo Scientific) supplemented with Halt™ Protease and Phosphatase Inhibitor Cocktail (78442; Thermo Scientific) for 15 min at 4 °C. The lysates were separated using 12% sodium dodecyl sulfate–polyacrylamide gel electrophoresis and electrotransferred onto a polyvinylidene fluoride membrane (Millipore Corporation, Billerica, MA, USA). The resulting blot was washed twice in Tris-buffered saline containing 0.1% Tween 20 (TBS-T), blocked in EveryBlot Blocking Buffer (12010020; Bio-Rad, Hercules, CA, USA) for 5 min, and then incubated with the primary antibodies overnight at 4 °C. After washing with TBS-T buffer, the membranes were incubated with horseradish peroxidase-conjugated secondary antibody (anti-rabbit, 1:3000) for 1.5 h at RT. Following subsequent washing with TBS-T, protein bands were visualized using a Lumino Graph II (ATTO, Tokyo, Japan) after incubation with SuperSignal™ West Pico PLUS Chemiluminescent Substrate (34582; Thermo Scientific). The antibodies used in this study are detailed in Supplementary Table 1.

### Statistical analysis

Statistical analysis was performed using SPSS 21.0 (SPSS Inc., Chicago, IL, USA). All experiments were repeated at least in triplicates and data were presented as the mean ± SEM. The significance between two groups was analyzed using Student’s t-test, while the significance between more than two groups was performed using one-way analysis of variance. Statistical significance was considered at *p* < 0.05.

## Electronic supplementary material

Below is the link to the electronic supplementary material.

Supplementary Material 1

## Data Availability

The datasets generated and analyzed during the current study are available from the corresponding author upon reasonable request.
