# Supplementary material for: Interleukin-7 promotes porcine early embryogenesis in vitro and inner cell mass development through PI3K/AKT pathway after parthenogenetic activation
Source: Sci Rep. 2025 Apr 22;15:13850. doi: 10.1038/s41598-025-98574-z (PMC12015589; doi:10.1038/s41598-025-98574-z)
Supplement: Supplementary file 1 — Supplementary Material 1 [file 41598_2025_98574_MOESM1_ESM.docx]

**Supplementary Information**

**Interleukin-7 promotes porcine early embryogenesis in vitro and inner cell mass development through PI3K/AKT pathway after parthenogenetic activation**

Dongjin Oh^1,2^, Hyerin Choi^1,2^, Mirae Kim^1,2^, Ali Jawad^1,2^, Joohyeong Lee^1,2,3^, Byoung Chol Oh^4^ and Sang-Hwan Hyun^1,2,5,6*^

^1^Laboratory of Veterinary Embryology and Biotechnology (VETEMBIO), Veterinary Medical Center and College of Veterinary Medicine, Chungbuk National University, Cheongju, South Korea

^2^Institute of Stem Cell and Regenerative Medicine (ISCRM), Chungbuk National University, Cheongju, South Korea

^3^Department of Companion Animal Industry, Semyung University, Jecheon 27136, Republic of Korea

^4^Department of Plastic and Reconstructive Surgery, Johns Hopkins University School of Medicine, Baltimore, MD, USA

^5^Vet-ICT Convergence Education and Research Center (VICERC), Chungbuk National University, Cheongju, Republic of Korea

^6^Chungbuk National University Hospital, Cheongju, Republic of Korea

***Corresponding authors:** Sang-Hwan Hyun, shhyun@cbu.ac.kr

**The Supplementary Information includes:**

Supplementary Figure 1

Original blots images for Supplementary Figure 1

Supplementary Tables 1 to 2


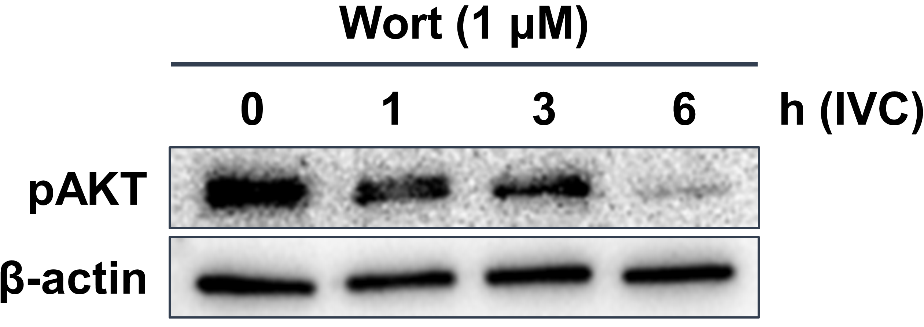


**Supplementary Figure 1.** Effect of wortmannin (Wort) treatment during *in vitro* culture (IVC) on phosphorylated AKT (pAKT) expression in embryos at specified time points after parthenogenetic activation.

**Original blots images for Supplementary Figure 1**

**pAKT**

**
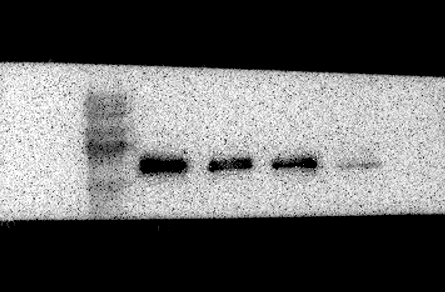
**

**β-actin**

**
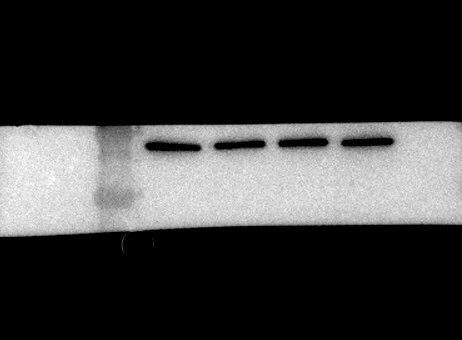
**

Supplementary Table 1. Antibodies used in this study.

| Reagent | Source | Cat # | Dilution |
| --- | --- | --- | --- |
| Mouse monoclonal anti-SOX2 | Santa Cruz Biotechnology | sc-365823 | 1:100 |
| Rabbit polyclonal anti-Phospho-AKT (Ser473) | Cell Signaling Technology | 9271S | 1:50 (For IF)  1:1000 (For WB) |
| Rabbit monoclonal anti-Phospho-S6 Ribosomal Protein (Ser235/236) | Cell Signaling Technology | 4858S | 1:100 |
| Rabbit polyclonal anti-β-actin | Cell Signaling Technology | 4967S | 1:1000 |
| Goat anti-Mouse IgG (H+L) Highly Cross-Adsorbed Secondary Antibody, Alexa Fluor™ 488 | Invitrogen | A-11029 | 1:400 |
| Donkey anti-Rabbit IgG (H+L) Highly Cross-Adsorbed Secondary Antibody, Alexa Fluor™ 594 | Invitrogen | A-21207 | 1:400 |

IF, Immunofluorescence; WB, Western blot

Supplementary Table 2. Primer sequences used for qRT-PCR.

| **Gene** | **Primer sequences (5'→3')** | **Product size (bp)** | **GenBank**  **accession number** |
| --- | --- | --- | --- |
| *RN18S* | F: CGCGGTTCTATTTTGTTGGT | 219 | NR_046261.1 |
|  | R: AGTCGGCATCGTTTATGGTC |  |  |
| *BAX* | F: TGCCTCAGGATGCATCTACC | 199 | XM_013998624.2 |
|  | R: AAGTAGAAAAGCGCGACCAC |  |  |
| *CASP3* | F: CGTGCTTCTAAGCCATGGTG | 186 | NM_214131.1 |
|  | R: GTCCCACTGTCCGTCTCAAT |  |  |
| *BCL2L1* | F: AATGACCACCTAGAGCCTTG | 182 | NM_214285.1 |
|  | R: GGTCATTTCCGACTGAAGAG |  |  |
| *MCL1* | F: GGACATCAAAAACGAAGACG | 181 | NM_001348806.1 |
|  | R: TGTGATGCTTTCTGCTAACG |  |  |
| *NANOG* | F: TAAAACCACTGCCCACATCT | 131 | NM_001129971.1 |
|  | R: CTGCCTCTGAAATCTGTCGT |  |  |
| *SOX2* | F: CCGTGGTTACCTCTTCTTCC | 186 | NM_001123197.1 |
|  | R: AGAGAGGCAGTGTACCGTTG |  |  |
| *KLF4* | F: GGCAAAACCTACACGAAGAG | 215 | NM_001031782.2 |
|  | R: ATGTGTAAGGCAAGGTGGTC |  |  |
| *KLF17* | F: GGGCAGCAGTTCAGTATGTC | 206 | NM_001164010.1 |
|  | R: GGCATCCTTAGATTCCCACT |  |  |
| *PDGFRA* | F: TGGAAACAGAAACCCAGGTA | 194 | NM_001315756.1 |
|  | R: TAGGCAGTTCCTTCAACCAC |  |  |
| *COL4A1* | F: CACAGTCAAACCACAGACGA | 180 | XM_021065910.1 |
|  | R: GACGTTGTTGATGTTGCAGA |  |  |
| *GATA3* | F: CTGTGCAAACTGTCAAACCA | 190 | NM_001044567.1 |
|  | R: GTGCACCTTTTTGCACTTTT |  |  |
| *DAB2* | F: ACAATGGGGTGTCTGAAAGA | 187 | XM_021076649.1 |
|  | R: CCTGGTTTGGTACTTTGTGG |  |  |
| *TFAM* | F: TGGTCCATCACAGGTAAAGC | 209 | NM_001130211.1 |
|  | R: CCTCAGTGTCTTTCTTTGCTG |  |  |
| *POLG* | F: GAGAAAGTCACGACGGAAGA | 183 | XM_001927064.5 |
|  | R: GTCATAAACTCCCCCTGGAC |  |  |
| *NRF1* | F: CCCAACCCTGTCTTTAAGGT | 187 | XM_021078993.1 |
|  | R: GGGTCATTTTGTCCACAGAG |  |  |
| *PPARGC1A* | F: AGCGAAGAGCATTTGTCAAC | 184 | NM_213963.2 |
|  | R: GGCTTGTAAATGTTGCGACT |  |  |

F: Forward, R: Reverse.
